# Epigenetic Regulation of Juvenile-to-Adult Transition in Plants

**DOI:** 10.3389/fpls.2018.01048

**Published:** 2018-07-17

**Authors:** Yunmin Xu, Lu Zhang, Gang Wu

**Affiliations:** State Key Laboratory of Subtropical Silviculture, School of Agriculture and Food Sciences, Zhejiang Agriculture and Forestry University, Hangzhou, China

**Keywords:** epigenetic regulation, miR156, SPL, juvenile-to-adult transition, plants

## Abstract

Epigenetic regulation is referred to as changes in gene function that do not involve changes in the DNA sequence, it is usually accomplished by DNA methylation, histone modifications (repressive marks such as H3K9me, H3K27me, H2Aub, or active marks such as H3K4me, H3K36me, H3Ac), and chromatin remodeling (nucleosome composition, occupancy, and location). In plants, the shoot apex produces different lateral organs during development to give rise to distinguishable phases of a juvenile, an adult and a reproductive phase after embryogenesis. The juvenile-to-adult transition is a key developmental event in plant life cycle, and it is regulated by a decrease in the expression of a conserved microRNA-miR156/157, and a corresponding increase in the expression of its target genes encoding a set of plant specific SQUAMOSA PROMOTER BINDING PROTEIN-LIKE (SPL) proteins. Recent work has revealed that the miR156/157-SPL pathway is the master regulator of juvenile-to-adult transition in plants, and genes in this pathway are subjected to epigenetic regulation, such as DNA methylation, histone modifications, and chromatin remodeling. In this review, we summarized the recent progress in understanding the epigenetic regulation of the miR156/157-SPL pathway during juvenile-to-adult transition and bring forward some perspectives of future research in this field.

## Introduction

Unlike mammals, in which organ formation is completed during embryonic development, plants produce new organs from self-sustaining stem cell populations known as meristems in different developmental processes. In plants, post-embryonic development can be divided into a juvenile vegetative phase, an adult vegetative phase and a reproductive phase, and each developmental phase is marked by changes in a series of distinct phase-specific traits (Poethig, 1990; Kerstetter and Poethig, 1998). The transition from the juvenile vegetative phase to the adult vegetative phase was referred to as the juvenile-to-adult transition or vegetative phase change.

In *Arabidopsis*, the juvenile-to-adult transition is characterized by the formation of leaf abaxial trichomes, an increase in leaf length/width ratio and serration, and a decrease in cell size (Telfer et al., 1997; Tsukaya et al., 2000; Usami et al., 2009). Genetic and molecular analyses demonstrated that the conserved miRNA-miR156/157 and its target genes-*SQUAMOSA PROMOTER BINDING PROTEIN-LIKE* (*SPL*) genes act sequentially with miR172, another miRNA that targets a class of *AP2*-like transcription factors (TFs), to regulate juvenile-to-adult transition in plants (Wu and Poethig, 2006; Wu et al., 2009; He et al., 2018). miR156/157 is highly expressed in juvenile phase and its abundance declines gradually, while its target *SPL* genes increases during shoot development. miR156/157 negatively regulates *SPL* gene expression through transcript cleavage or translational inhibition. *SPLs* were also responsive to photoperiodic induction and exhibited an miR156/157-independent expression pattern (Schmid et al., 2003; Jung et al., 2012). Therefore, the outcome of SPL levels fine-tuned by both miR156/157 and exogeneous cues orchestrates the timing of juvenile-to-adult transition (Huijser and Schmid, 2011; Poethig, 2013).

The Arabidopsis genome encodes eight miR156 genes (*MIR156A∼H*) and four miR157 genes (*MIR156A∼D*), and those genes function redundantly. The *mir156a mir156c* double mutant exhibited a similar phenotype to the *35S::MIMICRY156* transgenic plants with significantly reduced levels of miR156, which indicates that *MIR156A* and *MIR156C* are the two main loci contributing to the level of miR156 and have dominant roles in vegetative phase change within the miR156 family in *Arabidopsis* (Yang L. et al., 2013; Yu et al., 2013). miR157 functions redundantly with miR156, but has a much smaller effect on shoot morphology and *SPL* gene expression than miR156 (He et al., 2018). miR156/157 targets 10 out of 16 different *SPL* genes in *Arabidopsis*. Based on the amino acid sequence of the SBP domain, the miR156/157-targeted *SPL* genes can be classified into five clades, *SPL3/SPL4/SPL5*, *SPL9/SPL15*, *SPL2/SPL10/SPL11*, *SPL6*, and *SPL13A/B* (Xie et al., 2006; Riese et al., 2007; Preston and Hileman, 2013). Genetic and functional analysis of the role of *SPL* genes in vegetative phase change indicated that *SPL2/SPL9/SPL10/SPL11/SPL13/SPL15*, but not *SPL3/4/5/6*, contribute to the juvenile-to-adult transition with *SPL9/SPL13/SPL15* being more important for juvenile-to-adult transition than *SPL2/SPL10/SPL11* (Xu et al., 2016a).

**FIGURE 1:**
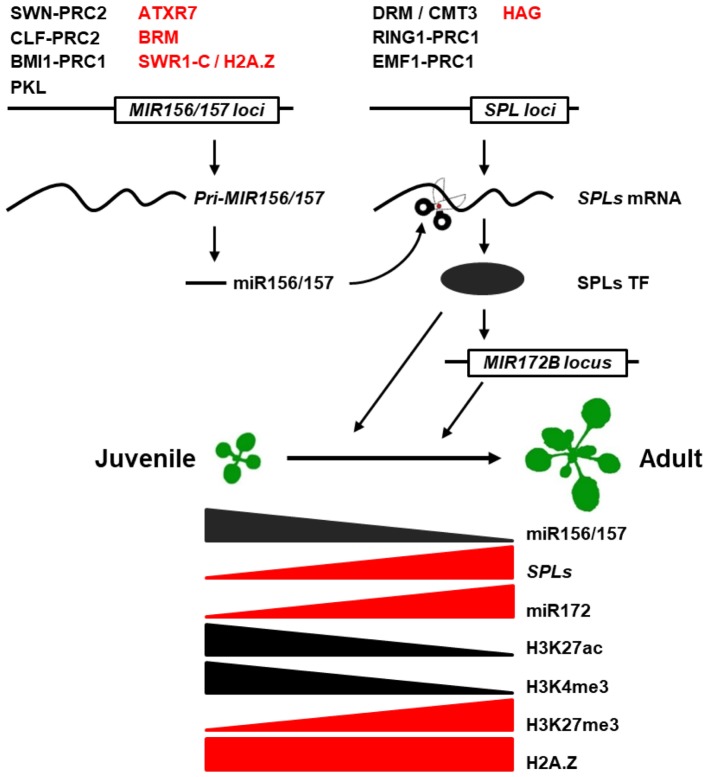
Epigenetic regulation of juvenile-to-adult transition in plants. Active and repressive epigenetic regulators were marked in red and black at *MIR156*/*157* and *SPL* loci, respectively. Triangle indicates gradual increase or decrease in the epigenetic modification levels of *MIR156* loci.

As the master regulator of the juvenile-to-adult transition, miR156/157-SPL pathway has been shown to be subjected to transcriptional and post-transcriptional regulation. Those include the transcriptional regulation of *pri-MIR156/157* and *SPLs* genes, the regulation of miR156/157 biogenesis, and post-transcriptional regulation of *SPL* genes (**Figure 1**). Here, we review our current understanding of epigenetic regulation of the miR156/157-SPL pathway and the roles of corresponding players in juvenile-to-adult transition in plants.

## DNA Methylation

DNA methylation [5-Methylcytosine (5mC)] is a hallmark of epigenetic gene silencing in both plants and mammals (Feng et al., 2010; Law and Jacobsen, 2010). DNA methylation is found at CG or non-CG sites including CHH and CHG (H represents A, T, or C) in plants in contrast to CG sites only in mammals (Henderson and Jacobsen, 2007; Cokus et al., 2008). In plants, CG methylation is carried out by DNA METHYLTRANSFERASE 1 (MET1), whereas DOMAINS-REARRANGED METHYLTRANSFERASEs (DRM) and CHROMOMETHYLASE 3 (CMT3) are responsible for the non-CG methylation (Law and Jacobsen, 2010).

The first indication of DNA methylation plays a role in phases of shoot development comes from the work done by Brink. In the 1950s, Brink noticed the similarity between phase change in plants and changes in cell states in non-plant organisms, he proposed that phases of shoot development might be regulated by reversible changes in chromatin based on his research on paramutation in maize (Brink, 1962). Subsequent work on *Spm* transposable elements (Banks and Fedoroff, 1989) and the Robertson’s Mutator (*Mu*) element (Martienssen et al., 1990) suggest that DNA methylation may be the underlying mechanism for maintaining phases of shoot development in plants. Recent work in peach also demonstrated that levels of nuclear DNA methylation was higher in adult meristems than that in juvenile and juvenile-like meristems (Bitonti et al., 2002), and an increase in DNA methylation during development seems widespread in plants (Fraga et al., 2002; Ruiz-García et al., 2005). In *Arabidopsis*, the triple DNA methyltransferase mutant *drm1 drm2 cmt3* exhibited a developmental retardation phenotype (Cao and Jacobsen, 2002), indicating that DNA methylation is important for normal growth and development in plants. However, genome-wide DNA methylation analysis of 5-week-old Columbia wild type, *met1* and *drm1 drm2 cmt3* triple mutant (Zhang et al., 2006), and 25-day-old Columbia wild type (Zilberman et al., 2007) indicated that only the coding sequence *of* the *SPL10* gene contains non-CG methylation. These results suggest that genes upstream or downstream of the miR156/157-SPL pathway, instead of miR156/157 or *SPL* genes, might be regulated by DNA methylation. Therefore, phenotypic characterization of vegetative phase change phenotype of mutants of DNA methyltransferases (MET1, DRM, and DNMT2) or demethylation enzymes (ROS1, DME, DML2, and DML3), as well as bisulfite sequencing of *MIR156*/*157* and *SPLs* loci, will facilitate to uncover the role of DNA methylation in regulation of miR156/157-SPL pathway and juvenile-to-adult transition in plants.

## Histone Modification

Histone modification at specific lysine sites functions as transcription repressive marks such as H3K9me, H3K27me, H2Aub, etc., or active marks such as H3K4me, H3K36me, H3Ac, etc., this modification is catalyzed by Polycomb group (PcG) protein complexes and Trithorax group (TrxG) protein complexes, respectively (Pien and Grossniklaus, 2007; Köhler and Hennig, 2010; Grossniklaus and Paro, 2014; Kingston and Tamkun, 2014). PcG complexes are repressors of gene transcription, and function in multi-subunit complexes, such as Polycomb Repressor Complex 1 (PRC1) or Polycomb Repressor Complex 2 (PRC2) (Grossniklaus and Paro, 2014).

## PRC2 and H3K27me3 Modification

PRC2 is a highly conserved and well-characterized PcG complex, and it represses target gene expression by trimethylating histone H3 at lysine 27 (H3K27me3) through the E(z) SET domain (Köhler and Hennig, 2010; Grossniklaus and Paro, 2014). In the Arabidopsis genome, three paralogous genes *MEDEA* (*MEA*), *SWINGER* (*SWN*), and *CURLY LEAF* (*CLF*) are orthologs of the *Drosophila E(z)* gene, which function as a histone methyltransferase subunit in the PRC2 complex. *MEA* appears to function in embryogenesis specifically, and *CLF* and *SWN* are broadly expressed and partially redundant in vegetative and reproductive development (Zheng and Chen, 2011; Bemer and Grossniklaus, 2012; Xu et al., 2016b).

Whole genome analysis in *Arabidopsis* uncovered 1000s of gene loci carrying the H3K27me3 mark catalyzed by the PRC2 complex, indicating that H3K27me3 is a major epigenetic silencing mechanism in plants (Zhang et al., 2007; Lafos et al., 2011). Among them, most *MIR156/157* loci, especially the dominant loci (*MIR156A*, *MIR156C*, and *MIR157A*), also carry H3K27me3 mark. However, except for *SPL4* and *SPL6* which play no obvious roles in juvenile-to-adult transition, miR156/157-targeted *SPL* genes are largely devoid of the H3K27me3 mark. These results imply that the PRC2 complex promotes *SPL* gene transcription indirectly by repressing the transcription of *MIR156/157* loci (Lafos et al., 2011).

During juvenile-to-adult transition in *Arabidopsis*, the decrease in the transcription of *MIR156A* and *MIR156C* loci is associated with an increase in the binding of the PRC2 complex to these two loci, causing an increase in the H3K27me3 mark in their promoter and transcribed regions as well as a decrease in the H3K27ac mark in the region immediately after transcription start sites (TSS) (Xu et al., 2016b,c). Loss-of-function mutant of *SWN*, but not the loss-of-function mutant of *CLF*, exhibited an obvious delayed juvenile-to-adult transition phenotype (Xu et al., 2016b,c). H3K27me3 was completely lost in *clf swn* double mutant and it eventually dedifferentiated into a callus-like tissue, making it impossible to determine the phenotype of juvenile-to-adult transition (Xu et al., 2016b). Therefore, the question of if *SWN* and *CLF* functions redundantly in vegetative phase change remains unknown. However, the H3K27me3 mark at *MIR156A/MIR156C* loci was significantly reduced in *clf* mutants, but that in *swn* mutant remains controversial, which indicates that *SWN* and *CLF* may function redundantly to repress *MIR156A/MIR156C* by catalyzing H3K27me3 (Xu et al., 2016b,c).

## PRC1 and Histone Ubiquitination

PRC1 is thought to recognize the H3K27me3 mark to confer stable transcriptional repression (Lund and van Lohuizen, 2004). PRC1 is more dissimilar between *Arabidopsis* and animals, but it has related functions. In *Arabidopsis*, the function of PRC1 can be histone 2A mono-ubiquitination (H2Aub) dependent or independent. H2Aub dependent group requires the E3 ubiquitin ligase activity of Arabidopsis B lymphoma Moloney murine leukemia virus insertion region1 homolog 1A (AtBMI1A)/B/C or AtRING1A/B, while H2Aub independent group requires the activity of the EMBRYONIC FLOWER 1 (EMF1) (Yang C. et al., 2013; Calonje, 2014). BMI1-PRC1 and RING1-PRC1 are required for the repression of seed maturation program after germination, whereas EMF1-PRC1 is required for floral repression (Moon et al., 2003; Calonje et al., 2008; Chen et al., 2010).

PRC1 has been shown to be involved in juvenile-to-adult transition in *Arabidopsis*. BMI1-PRC1 maintains the repression of miR156 and accelerates juvenile-to-adult transition (Picó et al., 2015). The levels of *MIR156A* and *MIR156C* were upregulated in *atbmi1a/b* mutant and the juvenile phase was prolonged with the H2Aub and H3K27me3 marks being decreased in the TSS region of *MIR156A* and *MIR156C* (Picó et al., 2015).

RING1-PRC1 and EMF1-PRC1 function to repress *SPLs* to delay juvenile-to-adult transition (Li et al., 2017). In *ring 1a ring 1b* double mutant, the H2Aub mark was obviously decreased in the promoter and coding region of *SPL3*, *SPL9* and *SPL10*, causing upregulation of these genes to accelerate the appearance of adult traits (Li et al., 2017). Therefore, PRC1 variants function in vegetative phase change mainly by targeting different *MIR156/157* loci or *SPL* genes in the miR156/157-SPL pathway, and they have opposing roles in this process. However, how PRC1 variants recognize distinct targets still remains unclear, and more work is required to explore the mechanism of how PRC1 works.

## ATXR7 and H3K4me3 Modification

The Arabidopsis genome encodes three H3K4 methyltransferase, namely ARABIDOPSIS TRITHORAX1 (ATX1), ATX2, and ATXR7 (Avramova, 2009). ATX1 and ATX7 are members of the Trithorax family, and ATXR7 is the only member of the SET1 subfamily in *Arabidopsis* (Tamada et al., 2009). *atxr7-1*, but not *atx1-1*, *atx2-1*, or *atx1 atx2* double mutant, exhibits a precocious juvenile-to-adult transition phenotype. Chromatin immunoprecipitation (ChIP) analyses indicated that ATXR7 binds to a region adjacent to the TSS of *MIR156A* and deposits the H3K4me3 mark to activate *MIR156A* transcription (Xu et al., 2018).

## HAG1 and Histone Acetylation

Histone acetylation is generally considered as an active epigenetic mark, which is a balanced process regulated by histone acetyltransferases (HAG1) and histone deacetylases (HDA1, HAD6). Spt–Ada–Gcn5–acetyltransferase-like histone acetyltransferase complex (SAGA-like complex) is conserved in mammals, plants, files and yeast, and General Control Non-repressed 5 (GCN5) functions as the catalytic component for this complex (Turner, 2000).

In *Arabidopsis*, loss-of-function mutants in *HAG1* (the Arabidopsis homolog of *GCN5*), *hag1-6* and *hag1-7*, exhibited a significantly delayed juvenile-to-adult transition phenotype (Kim et al., 2015). In *hag1-6* mutant, transcripts of *MIR156* loci and mature miR156 remained stable; however, those of *SPL3*, *SPL4*, *SPL5*, *SPL9*, *SPL11*, *SPL13*, *SPL15*, and *SPL8* were greatly reduced, suggesting that the regulation of *SPLs* by HAG1 is independent of miR156. ChIP results showed HAG1 was bound to the promoters and transcribed regions of *SPL3* and *SPL9* directly, leading to histone acetylation at the H3K9, H3K14, and H3K27 sites in these genes (Kim et al., 2015). HAG1-mediated H3 acetylation (H3Ac) of *SPL9* is also responsive to light signals, which indicates that HAG1-mediated H3Ac of *SPL9* might function as a sensor of environmental conditions to modulate the developmental process in plants (Kim et al., 2015).

## Chromatin Remodeling

Chromatin remodeling includes changes in nucleosome composition, nucleosome occupancy, nucleosome location, and the accessibility of the DNA to other transcriptional regulators.

## SWR1-C and H2A.Z Histone Variant

ATP-dependent SWR1 chromatin remodeling complex (SWR1-C) functions in exchanging the histone H2A-H2B dimer with the H2A.Z-H2B dimer, and then produces nucleosome variant (Mizuguchi et al., 2004; Luk et al., 2010). In *Arabidopsis*, mutations in the SWR1-C subunit coding genes (*ARP6*, *SEF*, and *PIE1*) and H2A.Z coding genes (*HTA8*, *HTA9*, and *HTA11*) exhibited a similar pleiotropic phenotype, which indicates that the primary function of SWR1-C is to deposit H2A.Z (Mizuguchi et al., 2004; Wu et al., 2005). However, the mechanism of H2A.Z modification by SWR1-C to regulate different target gene expression is distinguishable in that H2A.Z can change the nucleosome occupancy to destabilize nucleosomes or to increase nucleosome stability and/or to function with H3K4me3 mark together (Martin-Trillo et al., 2006; Kumar and Wigge, 2010; Choi et al., 2013).

In *arp6* and *hat9/hat11* mutants, *MIR156A/MIR156C* transcripts were reduced and juvenile-to-adult transition was accelerated (Choi et al., 2016; Xu et al., 2018). ChIP with H2A.Z antibody showed that H2A.Z was enriched at the first 500 nucleotides after TSS of *MIR156A/MIR156C*, and the level of H2A.Z was significantly reduced in *arp6* mutant. However, H2A.Z level does not change significantly during juvenile-to-adult transition, suggesting that H2A.Z and SWR1-C contribute to maintaining the expression of *MIR156A/MIR156C* early in shoot development, but do not regulate the timing of juvenile-to-adult transition (Xu et al., 2018). *MIR156A* transcript was reduced in *arp6* mutant due to higher nucleosome occupancy in its promoter region (Choi et al., 2016); however, it was suggested that H2A.Z increases the expression of *MIR156A/MIR156C* by promoting the deposition of H3K4me3 rather than by decreasing nucleosome occupancy in the *MIR156A* promoter region (Xu et al., 2018).

## ATP-Dependent Chromatin Remodeling Protein

BRAHMA (BRM) is the ATPase subunit of the most widely studied SWI2/SNF2 chromatin remodeling protein complex. It uses the energy derived from ATP hydrolysis to change the histone octamer-DNA interaction (Saha et al., 2006; Clapier and Cairns, 2009). BRM regulates *MIR156A* transcription by directly binding to the promoter region and maintaining low occupancy of the -2 and +1 nucleosomes proximal to the TSS. *brm* mutants exhibit an accelerated juvenile-to-adult transition phenotype by reducing the transcription of *MIR156A* (Xu et al., 2016c). BRM also antagonizes the function of SWN in the PRC2 complex to remove H3K27me3 repressive mark in *MIR156A* (Xu et al., 2016c).

PICKLE (PKL) is a CHD3 ATP-dependent nucleosome remodeling protein, which is physically associated with the nucleosome remodeling and deacetylation complex (Perruc et al., 2007; Zhang et al., 2008; Ho et al., 2013). PKL is bound to the TSS adjacent region of *MIR156A/MIR156C* to promote the juvenile-to-adult transition by repressing the transcription of *MIR156A/MIR156C*. In *pkl* mutants, *MIR156A/MIR156C* transcripts were elevated due to the reduction in nucleosome occupancy at the +1 position, an increase in the H3K27ac mark, and a corresponding decrease in the H3K27me3 mark in the promoter and transcribed region (Xu et al., 2016b).

## Perspective

**FIGURE 2:**
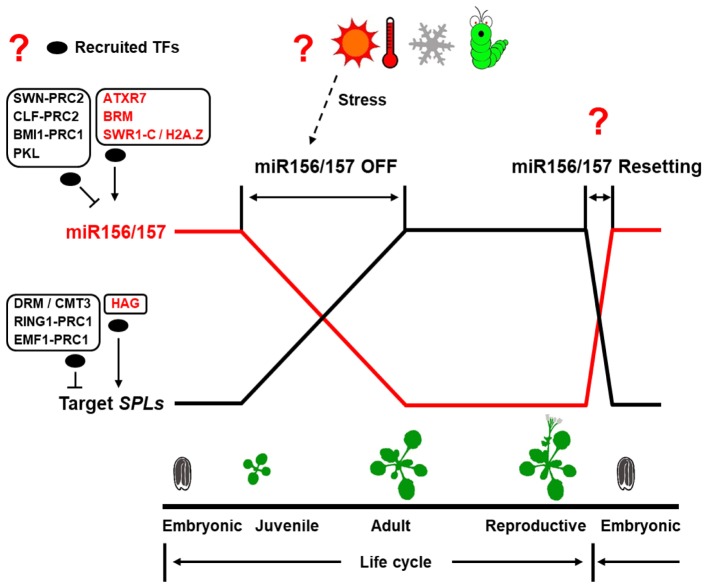
Epigenetic regulation of the miR156/157-SPL pathway in plant lifecycle. In plant lifecycle, the transcription of genes in the miR156/157-SPL pathway exhibits a fixed temporal expression pattern. The major unknown parts in epigenetic regulation of the miR156/157-SPL pathway are shown in question mark. Oval represents recruited transcription factors (TFs).

Although the miR156/157-SPL pathway has been shown to be the master regulator of juvenile-to-adult transition in plants, yet little is known about the upstream regulator of this pathway, especially for miR157. Recent studies have revealed that DNA methylation, histone modification, chromatin remodeling play important roles in regulating the expression of some components in the miR156/157-SPL pathway. However, there are still some critical questions remain to be solved as illustrated in **Figure 2**.

## How are Epigenetic Regulators Recruited to the *MIR156/157* and/or *SPLs* Loci?

*MIR156/157* and/or *SPL* loci are subjected to epigenetic regulation to modulate juvenile-to-adult transition in plants. However, these epigenetic regulators, by their own, have no DNA binding specificity. Therefore, a central question is how these epigenetic regulators are recruited to their target genes.

PRC2-mediated H3K27me3 is a conserved epigenetic modification between plants and the animal kingdom (Mozgova and Hennig, 2015; Xiao and Wagner, 2015). Recent genomic study in *Arabidopsis* showed that PRC2 components bind to specific DNA motifs called Polycomb response elements (PREs) by interacting with specific TFs (Xiao et al., 2017). Interestingly, six top enriched motifs (CTCC, CCG, G-box, GA repeat, AC-rich, and Telobox motifs) out of 170 computationally defined PREs were present at the *MIR156A* locus (Xiao et al., 2017). The GA repeat and Telobox motifs were present adjacent to the TSS region of *MIR156A* and *MIR156C* loci together, these motifs are the potential binding sites for class I BPC and C1-2iD TFs, respectively. This information will be helpful to identity TFs through which the PRC2 complex interacts to be recruited to the *MIR156A/MIR156C* loci during juvenile-to-adult transition.

## Epigenetic Modification of *MIR156/157* and/or *Spl* Loci by Stress?

Plants are sessile organisms and they are forced to adapt to the changing environment. The miR156/157-SPL pathway functions as the master regulator of juvenile-to-adult transition and flowering (Wang et al., 2009; Wu et al., 2009). Therefore, plants evolved a precise mechanism to adapt to the environment by shortening or prolonging the juvenile phase or changing the flowering time. Under salt or drought stress conditions, miR156 was induced to maintain plants in the juvenile phase for a relatively longer time; when they were returned to favorable conditions, miR156 was suppressed to accelerate the developmental transition (Cui et al., 2014). Under UV-B radiation conditions, the PRC2-mediated H3K27me3 modification in the *MIR156A/MIR156C* loci was decreased, and the corresponding up-regulation of miR156 delayed juvenile-to-adult transition (Dotto et al., 2018). Other studies also indicate that the expression of miR156 is responsive to ambient temperature (Stief et al., 2014), phosphate starvation (Hsieh et al., 2009), CO_2_ treatment (May et al., 2013), suggesting a tight interaction between juvenile-to-adult transition and environment through the miR156/157-SPL pathway.

Epigenetic modification is a reversible mark, which can be removed or deposited to target genes to affect their expression in response to changing environment. It will be of great interest to learn how epigenetic modification patterns of *MIR156/157* and/or *SPL* loci change in response to external cues, especially to environment stresses, as well as how this changing environment affects the juvenile-to-adult transition.

## Reversible Epigenetic Regulation of miR156/157 Resetting?

During juvenile-to-adult transition, miR156/157 transcription was reduced or silenced gradually to ensure the plant to enter the adult phase and flower. This is achieved by disposing of active epigenetic marks such as H3K4me3, H3K27ac and depositing some repressive epigenetic marks such as H3K27me3 to miR156 loci. Interestingly, this silencing process needs to be reset to an active state in each generation as miR156/157 is de-repressed again to be highly expressed in the pro-embryo stage (Nodine and Bartel, 2010) after flowering.

A similar example of Off-Resetting pattern in plant lifecycle is the regulation of *FLOWERING LOCUS C* (*FLC*). *FLC* is silenced by depositing H3K27me3 mark under winter cold treatment, and the silenced state was maintained in the mature pollen grains and the egg cells (De Lucia et al., 2008; Sheldon et al., 2008). In pro-embryo stage, *FLC* is activated by depositing active epigenetic marks such as H3K4me3, H3K36me3, and disposing of repressive marks such as H3K27me3. LEAFY COTYLEDON1 (LEC1), a seed-specific pioneer TF (Tao et al., 2017), and EARLY FLOWERING 6 (ELF6), a H3K27me3 demethylase (Crevillén et al., 2014), were shown to play critical roles in *FLC* re-activation.

As for *MIR156/157*, it is still unknown where and when the *de novo* re-activation occurs. Moreover, whether the resetting of miR156/157 depends on a reversible epigenetic regulation still remains elusive. Further study of when, where and how miR156/157 Off-Reset pattern is initiated during plant life cycle will be an important future task.

## Author Contributions

YX and LZ wrote the article, and GW revised it.

## Conflict of Interest Statement

The authors declare that the research was conducted in the absence of any commercial or financial relationships that could be construed as a potential conflict of interest.
